# Structure and function of GluN1-3A NMDA receptor excitatory glycine receptor channel

**DOI:** 10.1126/sciadv.adl5952

**Published:** 2024-04-10

**Authors:** Kevin Michalski, Hiro Furukawa

**Affiliations:** W.M. Keck Structural Biology Laboratory, Cold Spring Harbor Laboratory, Cold Spring Harbor, NY 11724, USA.

## Abstract

*N*-methyl-d-aspartate receptors (NMDARs) and other ionotropic glutamate receptors (iGluRs) mediate most of the excitatory signaling in the mammalian brains in response to the neurotransmitter glutamate. Uniquely, NMDARs composed of GluN1 and GluN3 are activated exclusively by glycine, the neurotransmitter conventionally mediating inhibitory signaling when it binds to pentameric glycine receptors. The GluN1-3 NMDARs are vital for regulating neuronal excitability, circuit function, and specific behaviors, yet our understanding of their functional mechanism at the molecular level has remained limited. Here, we present cryo–electron microscopy structures of GluN1-3A NMDARs bound to an antagonist, CNQX, and an agonist, glycine. The structures show a 1-3-1-3 subunit heterotetrameric arrangement and an unprecedented pattern of GluN3A subunit orientation shift between the glycine-bound and CNQX-bound structures. Site-directed disruption of the unique subunit interface in the glycine-bound structure mitigated desensitization. Our study provides a foundation for understanding the distinct structural dynamics of GluN3 that are linked to the unique function of GluN1-3 NMDARs.

## Teaser

Cryo-EM structures of agonist- and antagonist-bound GluN1-3A NMDARs unveil robust structural changes coupled to channel activity.

## INTRODUCTION

*N*-methyl-d-aspartate (NMDA) receptors (NMDARs) are ionotropic glutamate receptors (iGluRs) that mediate most of the excitatory neurotransmission in mammalian brains. The canonical NMDARs are tetrameric ligand-gated ion channels, requiring binding of glycine and glutamate for channel opening. Coincidental detection of glutamate transmission from the presynapses and membrane depolarization in the postsynapses result in the Ca^2+^ influx into the postsynapse, pivotally involved in synaptic plasticity for learning and memory formation. NMDARs are formed from two obligate GluN1 subunits and two of any GluN2(A-D) or GluN3(A-B) subunits. Each GluN2 or GluN3 subunit confers differential attributes to channel properties, including activation, deactivation and desensitization kinetics, pH sensitivity, Ca^2+^ permeability, and binding to allosteric modulators, such as zinc, polyamines, and phenylethanolamines (*1*, *2*).

The NMDAR subunits are modular and harbor tiered domains that function in concert to regulate opening and closing of the cation-selective ion channel pore. The extracellular amino-terminal domains (ATDs) are a site of allosteric regulation to modulate overall receptor function, the ligand-binding domains (LBDs) bind to glycine (GluN1, GluN3) and glutamate (GluN2) and control opening of the channel gate, the transmembrane domain (TMD) harbors the channel gate and pore, and the carboxyl-terminal domain (CTD) in the cytoplasmic facet regulates channel function and cellular signaling via protein-protein interactions and posttranslational modifications (*1*, *3*, *4*). Extensive structural biology efforts by the field have unveiled the architecture of GluN2-containing NMDARs, providing a wealth of mechanistic information regarding substrate recognition, channel activation and desensitization, and inhibition by competitive and allosteric compounds (*5*–*13*).

The GluN1-3 (A or B) NMDAR emerges as an exception, distinguished by its distinctive ion channel activation mechanism, which relies exclusively on glycine and does not involve glutamate (*14*, *15*). They conduct cations (Na^+^/K^+^) as in GluN1-2 NMDARs (*14*), making GluN1-3 NMDARs excitatory glycine receptor channel, in contrast to glycine’s conventional role as an inhibitory neurotransmitter via pentameric glycine receptor channels in spinal cord and brainstem (*16*). In these NMDARs, glycine binding to GluN3 promotes channel opening, whereas glycine binding to GluN1 triggers rapid desensitization (*14*, *17*, *18*). Notably, in GluN1-3A NMDARs expressed heterologously in cell culture, the desensitization can be mitigated by competitive antagonists targeting GluN1, such as CGP-78608, while glycine occupies GluN3A subunits (*19*, *20*). A similar GluN1-3 NMDAR current can be observed by applying CGP-78608 and glycine in hippocampal neurons, indicating that the GluN1-3 NMDARs exist in the central nervous system (*19*). The GluN1-3 NMDARs have been shown to play crucial biological roles, including regulation of fear-related memories in cortical and amygdala circuits in adult mice (*21*) and aversive states in medial habenula (*22*). This underscores their physiological role as mediators of an excitatory glycine signal (*23*). It is worth noting that the GluN3 subunit has been suggested to form triheteromeric NMDARs with GluN1 and GluN2 subunits, which are activated by both glycine and glutamate (*24*). These GluN3-containing NMDARs exhibit distinct properties, including low Ca^2+^ permeability and reduced sensitivity to channel blockers like Mg^2+^, memantine, and MK-801, which is in contrast to GluN2-containing NMDARs (*1*, *23*, *25*).

To date, our understanding of the structural characteristics of the GluN3A subunit has predominantly hinged on x-ray crystallography and molecular dynamic simulation investigations carried out on the isolated LBD (*26*, *27*). The previous study unveiled a clamshell-like fold in the GluN3A LBD, akin to other iGluRs where glycine binding to the bi-lobe interface leads to a modest 8° clamshell closure compared to the antagonist-bound conformation (*26*, *27*). However, how GluN3A assembles with GluN1 to form an ion channel and why glycine induces strong desensitization remain largely unknown. Here, we provide cryo–electron microscopy (EM) structures of the intact GluN1a-3A NMDAR channels in complex with an antagonist, cyanquixaline (CNQX), and an agonist, glycine. We demonstrate that GluN1a-3A NMDAR assembles as a 1-3-1-3 heterotetramer, and that binding of glycine induces robust ~80° rotations of the GluN3A LBDs. Finally, we show through electrophysiology that site-directed disruption of the GluN1-3A LBD interface unique to the glycine-bound structure mitigates desensitization, indicating that the unique conformation of the glycine-bound structure represents a desensitized state.

## RESULTS

### Characterization, production, and structural analysis of GluN1-3A NMDAR

**Fig. 1.**
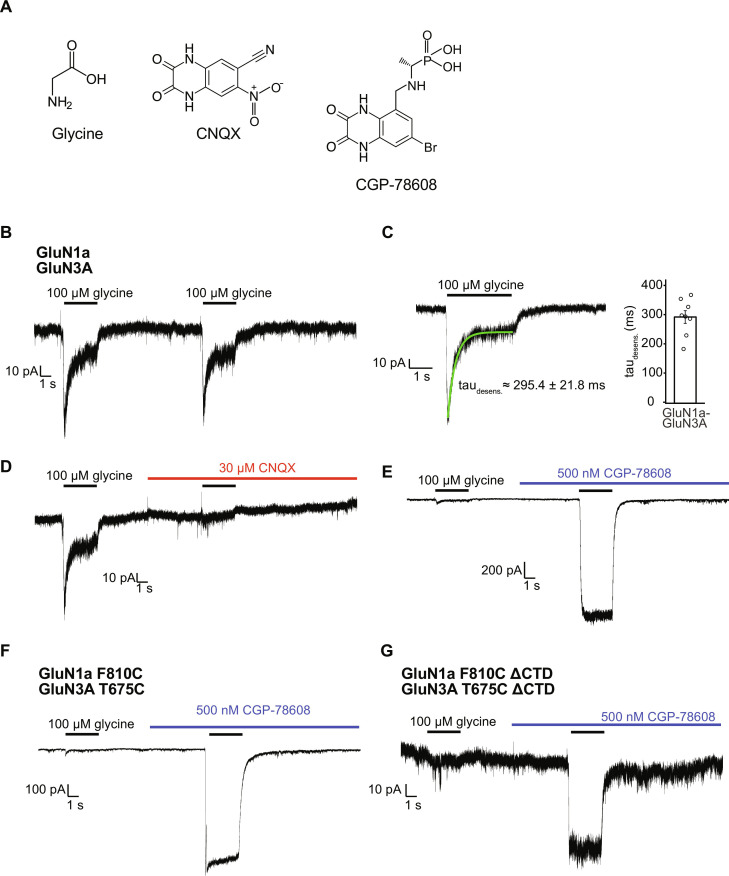
Electrophysiological characterization of GluN1a-3A NMDAR constructs. (**A**) Chemical structure of the GluN1a-3A NMDAR binding compounds. (**B**) Whole-cell patch-clamp recordings of HEK293T cells transfected to express WT human GluN1a and WT human GluN3A. Cells were held at −80 mV and exposed to 100 μM glycine for 3 s, with 10 s of rest between each application. (**C**) Example recording (left) demonstrating the extent of receptor desensitization during a 3-s treatment with glycine. The glycine response was fit with a single exponential (green curve), where the tau value was measured to be 295.4 ± 21.8 ms (mean ± SE). (**D**) Inhibition of WT GluN1a-3A NMDAR by CNQX. A solution containing 30 μM CNQX was perfused onto the cell for 5 s before exposure to a solution containing both CNQX and glycine. (**E**) Potentiation of GluN1a-3A NMDAR by CGP-78608. A solution containing 500 nM CGP-78608 was perfused onto the cell for 5 s before treatment with a solution containing both CGP-78608 and glycine. (**F**) Recording of the GluN1a Phe^810^Cys and GluN3A Thr^675^Cys mutants performed as described in (C). (**G**) Recording of the GluN1a Phe^810^Cys ΔCTD and GluN3A Thr^675^Cys ΔCTD mutants used in the cryo-EM studies. All recordings were performed on at least four different cells. A representative recording is shown for each experiment.

The wild-type (WT) human GluN1a-3A NMDAR displays rapidly desensitizing currents upon glycine stimulation (Fig. 1, A to C). These currents are inhibited by a competitive antagonist, CNQX, which binds to both the GluN1 and GluN3A LBDs (Fig. 1D) (*19*, *20*, *28*). Conversely, the glycine-activated currents are potentiated by a GluN1-selective antagonist, CGP-78608, by 25- to 50-fold (Fig. 1E) as reported previously (*19*, *29*).

The WT GluN1a-3A NMDAR protein sample displayed an instability that interfered with structure determination. Therefore, we sought to develop more stable expression construct amenable for single-particle cryo-EM while still maintaining intrinsic channel functions. Toward this end, we initially truncated GluN1a and GluN3A C-terminal residues after M4′ (fig. S1A) and coexpressed them in Sf9 insect cells using the EarlyBac method (*30*). The modified GluN1a-3A NMDAR had a yield and stability sufficient for structural analysis when purified in the detergent, LMNG, previously used in our studies on GluN2-containing NMDARs (*31*). A preliminary cryo-EM study on GluN1a-3A NMDAR in the presence of an antagonist, CNQX, resulted in a structure that had an NMDAR-like shape, but limited at low resolutions (~7 to 9 Å overall resolution). This low-resolution reconstruction suggested that GluN1a-3A NMDAR assembles into a heterotetrameric arrangement similar to GluN2-containing NMDARs. Previous x-ray and cryo-EM structures of GluN2-containing NMDARs used an engineered pair of cysteine mutations to form a stabilizing disulfide bond between GluN1a TM helix 4 and GluN2B TM helix 1 without altering channel functions (*6*, *31*). Here, we introduced the equivalent cysteine mutations into GluN1a (Phe^810^Cys) and GluN3A (Thr^675^Cys) to form the GluN1a-3A inter-subunit disulfide bond and stabilize the GluNa-3A NMDAR tetramer for potential improvement of the cryo-EM data (fig. S1, B and C). Furthermore, we found that the GluN1a-3A NMDAR proteins purified in digitonin and then exchanged into the amphipol, PMAL-C8, had reduced particle clumping under cryo-EM. The selected particles from this sample preparation resulted in reconstructions of GluN1a-3A NMDAR at sufficiently high resolution for molecular modeling. Whole-cell patch-clamp electrophysiology performed on transfected human embryonic kidney (HEK) 293 cells showed that the C-terminal truncation or introduction of the inter-subunit disulfide bond is inconsequential for channel activity, including inhibition by CNQX and potentiation by CGP-78608 (Fig. 1, F and G, and fig. S2) despite reduced current amplitudes likely due to altered surface expression.

### Structure of GluN1a-3A NMDAR in complex with an antagonist CNQX

**Fig. 2.**
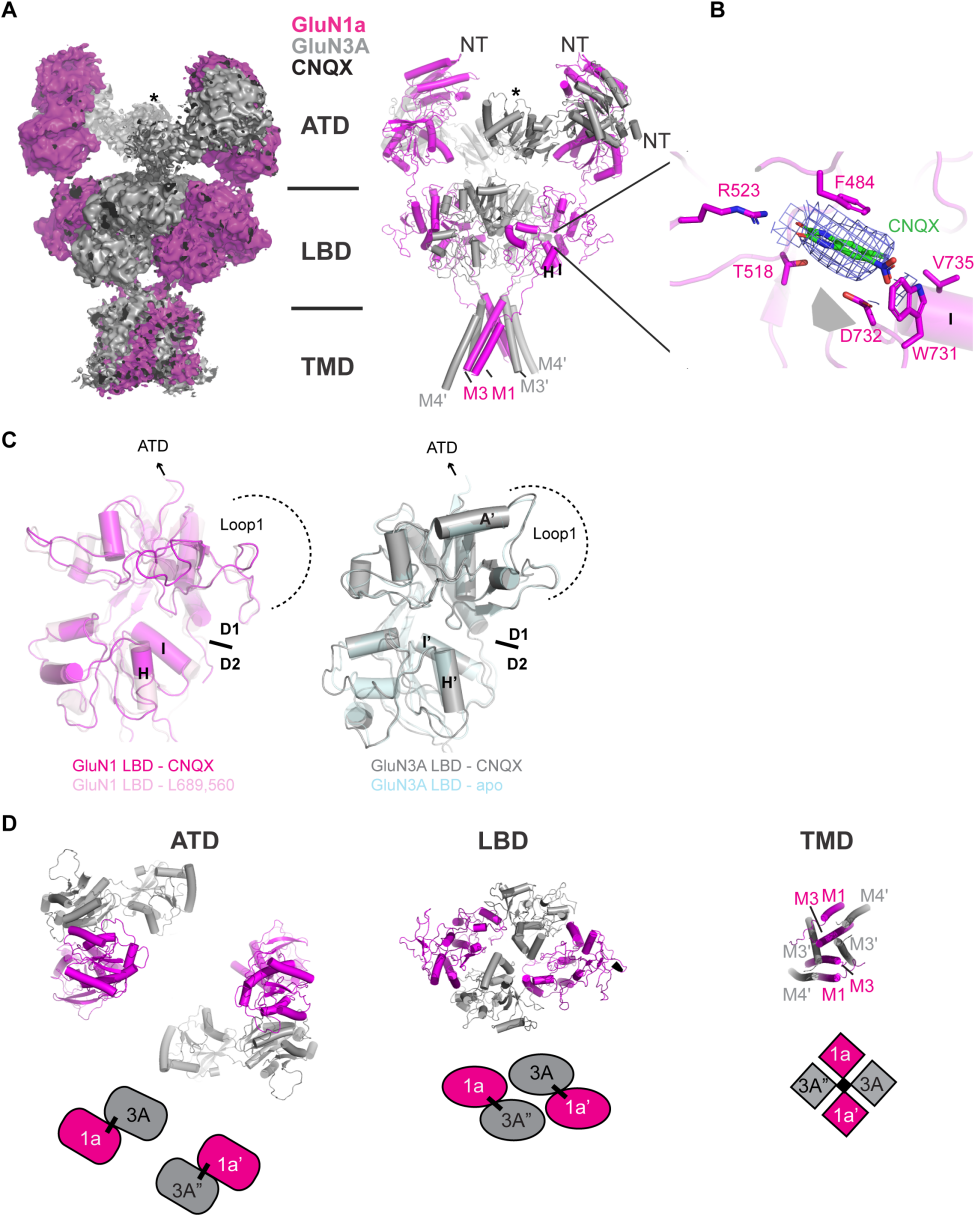
Cryo-EM structure of CNQX-bound GluN1a-3A NMDAR. (**A**) Cryo-EM density (left) and the fitted model. The density was sufficiently resolved for distinguishing GluN1a (magenta) and GluN3A (gray) subunits. GluN1a M4, GluN3A M1′, and the lower lobe of GluN3A ATD (asterisk) are not well resolved. (**B**) CNQX binding site at the cleft of the GluN1a LBD, showing the density (blue mesh) representing a CNQX molecule (green stick). (**C**) The GluN1 LBD and GluN3A LBD in the GluN1a-3A NMDAR structure have open cleft conformation similar to the L689,560-bound GluN1 LBD (left, RMSD = 1.15 Å over 267 Cɑ positions, PDB code: 6WHU) and the apo GluN3A LBD (right, RMSD = 0.85 Å over 254 Cɑ positions, PDB code: 4KCD). (**D**) Subunit arrangement of GluN1a and GluN3A viewed from the extracellular side. Two GluN1a-3A heterodimers (tethered by a black line) are arranged in the 1a-3A-1a′-3A′ pattern in each domain layer. Dimer pairs swap different between the ATD and LBD layers. The TMD channel pore is formed by the M3/M3′ helices.

We initiated our single-particle cryo-EM on GluN1a-3A NMDAR bound to a competitive antagonist, CNQX, to capture the inhibited state. Our analysis resulted in the structure to an overall resolution of 4.23 Å (Fig. 2, A and B; figs. S3 and S4; and table S1). The final reconstruction has the highest resolution at the LBD heterotetramer layer reaching up to ~3.8 Å locally (figs. S3 and S4). In contrast, the ATD layer has substantially weaker density, owing to flexibility of this domain with respect to the LBD and TMD layers (Fig. 2A and figs. S3 and S4). The two-dimensional (2D) class averages likewise indicated highly mobile ATDs juxtaposed beside the high-resolution features of the LBDs (figs. S3 and S4). This degree of ATD flexibility is not observed in GluN2-containing NMDARs, and attempts to resolve this domain at high resolution by local refinement or focused 3D classification were unsuccessful. Nevertheless, we could reliably observe the bi-lobe architectural features of the ATDs composed of R1 and R2 lobes (*32*–*37*) and the GluN1a-3A heterodimeric pairings. Thus, we could rigid-body fit the R1 and R2 models of the AlphaFold2 model (*38*) of the human GluN3A ATD separately into the weak cryo-EM density (Fig. 2, A and B). Furthermore, we could differentiate the GluN1a and GluN3A subunits with confidence due to featureful structural differences in the loop 1 region of GluN1 and GluN3A (Fig. 2C). This motif contains the 12-residue long helix A′, present in GluN3A LBD (*26*) but not in GluN1 LBD (*39*) (Fig. 2C). Moreover, the local resolution of the LBD layer is sufficiently high to resolve all residues with confidence (figs. S3 and S4). Hence, our analysis demonstrated that the GluN1-3A NMDAR heterotetramer is arranged with subunits in a 1-3-1-3 pattern, confirming that diheteromeric GluN1-3A receptors assemble as a “dimer of heterodimers” similar to GluN2-containing receptors (Fig. 2D) (*6*, *7*). As in other NMDAR, AMPA, and kainate receptors, we observe domain swapping between the ATD and LBD layers, and pseudo-fourfold symmetry in the central channel pore formed by the M3 helices (Fig. 2D) (*6*, *40*, *41*). The GluN3A subunits occupy the positions of the GluN2 subunit, which dominantly control gate opening and closure (Fig. 2D) (*5*, *10*). This overall topology is in contrast to GluD1 and GLR3.4 receptors, which display non–domain-swapped architecture (*42*, *43*).

We observe prominent density, consistent with CNQX, in the glycine binding pocket of the GluN1a LBD (Fig. 2B). The CNQX-bound GluN1a LBD has a similar open-cleft conformation to the GluN1 LBD bound to another antagonist, L689,560 [root mean square deviation (RMSD) = 1.15 Å over 267 Cɑ positions, Protein Data Bank (PDB) code: 6WHU] (Fig. 2C). While CNQX has been previously shown to bind the isolated GluN3A LBD (*28*), we do not detect a discernible CNQX density within the ligand-binding pocket of the GluN3A LBD. This absence may be attributed to low occupancy or poor local resolution. Nevertheless, a structural comparison with the apo-state or glycine-bound GluN3A LBD crystal structures (*26*, *27*) indicates that our GluN3A subunits also adopt an open-clamshell conformation similar to the apo-state (RMSD = 0.85 Å over 254 Cɑ positions, PDB code: 4KCD) (Fig. 2C). Stabilization of the open-cleft conformation within the LBDs represents inhibition in all iGluRs (*1*, *44*), including NMDARs (*45*, *46*), and is in line with our electrophysiology-based anticipation that CNQX would trap an inhibited state.

### Structure of glycine-bound GluN1-3A NMDARs shows unique subunit arrangement

We next sought to determine the structure of GluN1a-3A NMDAR in complex with glycine, an agonist that binds to both the GluN1a and GluN3A LBDs. We hypothesized based on the electrophysiological observation (Fig. 1, B and C) that prolonged exposure to 1 mM glycine during protein purification would promote a desensitized state. Thus, we anticipated uncovering the structural conformation of the desensitized GluN1a-3A NMDAR.

**Fig. 3.**
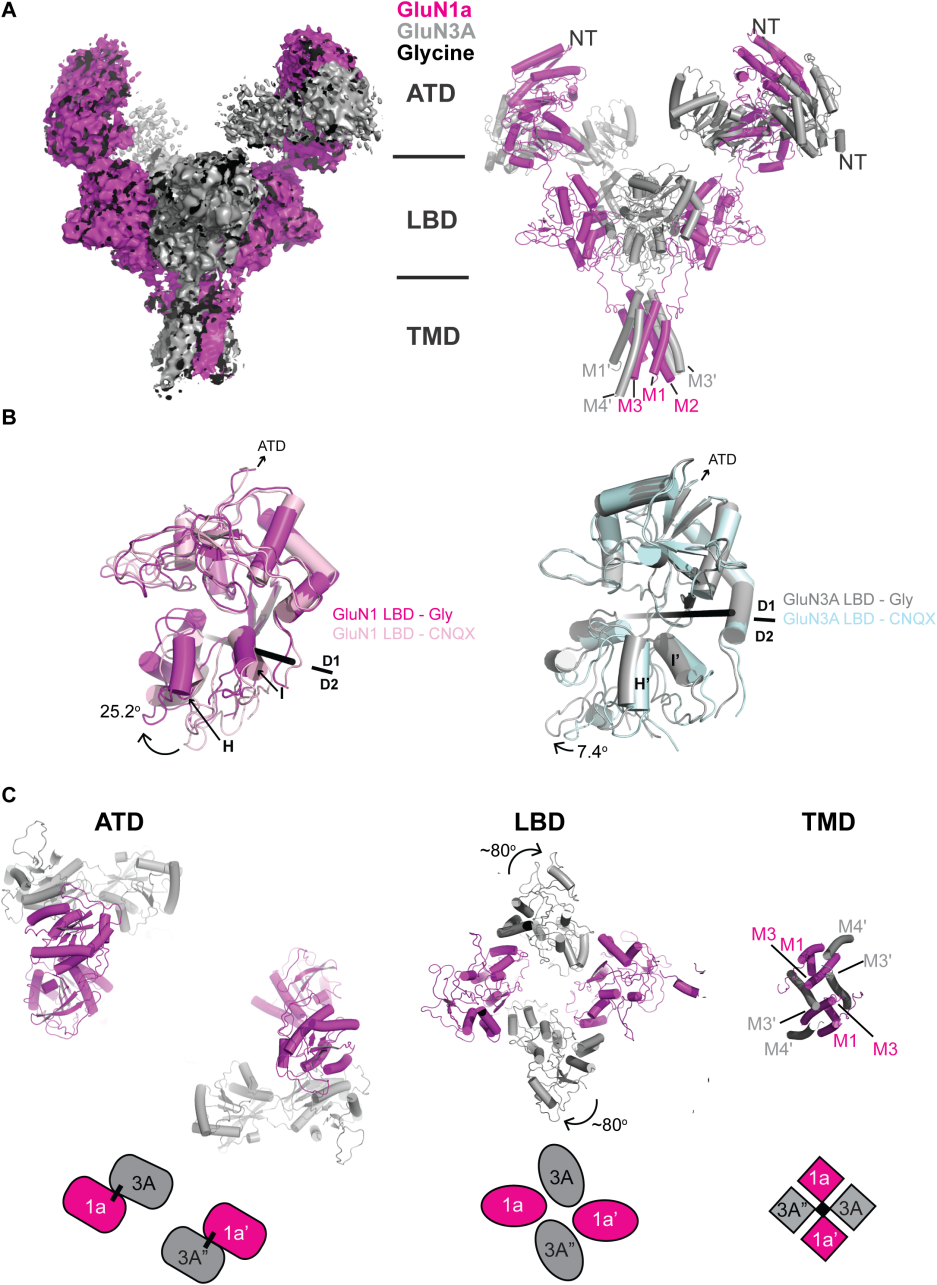
Cryo-EM structure of glycine-bound GluN1a-3A NMDAR. (**A**) Cryo-EM density (left) and the fitted model. The density was sufficiently resolved for distinguishing GluN1a (magenta) and GluN3A (gray) subunits. (**B**) Comparison of the glycine- and CNQX-bound GluN1a-3A NMDARs at LBDs. The upper lobe (D1) residues are superposed, and the rotation angles to align the D2 residues were calculated for GluN1a and GluN3A LBDs. The black rods represent rotational axes. (**C**) Subunit arrangement of GluN1a and GluN3A viewed from the extracellular side. Two GluN1a-3A heterodimers (tethered by a black line) are arranged in the 1a-3A-1a′-3A′ pattern in the ATD. The GluN1a-3A heterodimer interface is disrupted in LBDs due to ~80° clockwise rotation of the GluN3A subunits compared to the CNQX-bound structure. The TMD channel pore is formed by the M3/M3′ helices.

We obtained a reconstruction of glycine-bound GluN1a-3A NMDAR at 4.05 Å overall resolution by single-particle cryo-EM (Fig. 3A, figs. S5 and S6, and table S1). This structure shows the dimer of GluN1a-3A heterodimers in the 1-3-1-3 subunit arrangement as in the CNQX-bound GluN1a-3A NMDAR (Figs. 2 and 3). The ATD heterodimers are also highly mobile and only weakly resolved, indicating that the high degree of ATD mobility of GluN3A-containing receptors occurs independently of ligand occupancy within the LBD layer. We could resolve additional density of the TMD compared to the CNQX-bound structure, which allowed unambiguous registering of the GluN3A M3′ and M4′ helices.

Several key differences emerged within the LBD heterotetramer layer when comparing the glycine- and CNQX-bound structures. First, we observe closure of the GluN1a and GluN3A LBD bi-lobes. The degree of observed domain closure is consistent with previous studies on the isolated LBDs, which demonstrate that agonists stabilize a close-cleft LBD conformation (*26*). This suggests that our structure represents a glycine-bound configuration, although we cannot explicitly identify density for glycine in our reconstruction. Specifically, the glycine-bound GluN1a and GluN3A LBDs are closed by 25.2° and 7.4° compared to the CNQX-bound form, respectively (Fig. 3B). Second, the glycine-bound structure of GluN1a-3A NMDAR exhibits a pronounced conformational change within the LBD heterotetramer where the GluN3A LBDs undergo ~80° clockwise rotation when viewed from the extracellular side (Fig. 3C and movie S1). This robust GluN3A LBD rotation shift occurs around an axis orthogonal to the membrane plane. Such change in subunit orientation is previously unseen in any of the NMDAR subtypes.

### Comparison of GluN1-3A with GluN1-2B NMDAR

**Fig. 4.**
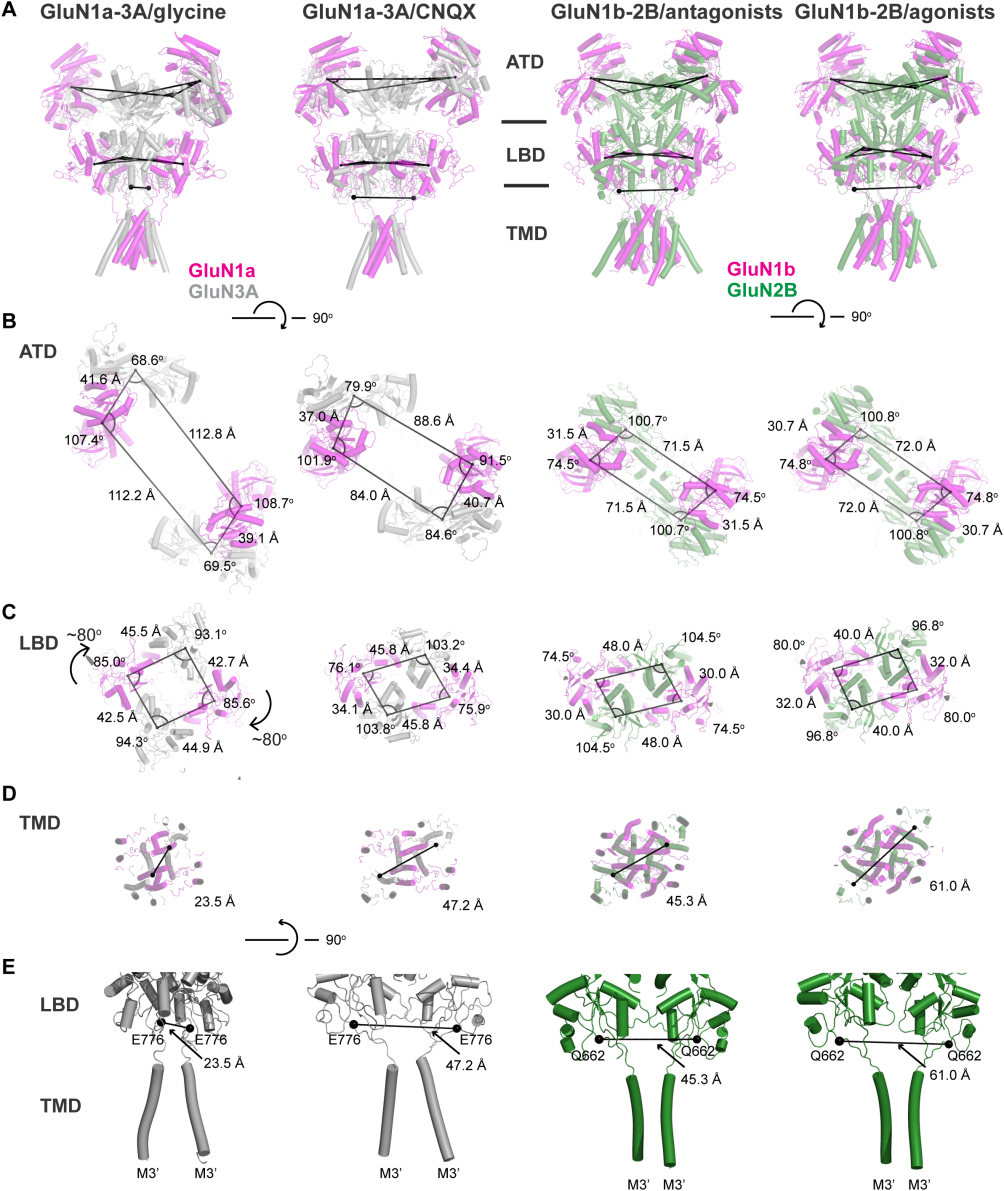
Comparison of GluN1-3A and GluN1-2B NMDARs in different states. (**A**) Side views of the receptors in different functional states. COM of ATDs and LBDs are connected by lines. At the bottom of the LBDs are GluN2B Gln^662^ and GluN3A Glu^776^, which are connected to the TMD channel and used to measure the LBD-TMD loop tension. (**B** and **C**) Top views of ATDs and LBDs where COMs are connected by lines and angles are measured, showing different subunit orientations. (**D** and **E**) Cɑs of GluN2B Gln^662^ and GluN3A Glu^776^ residues (spheres) connected to each other viewed from the top and side.

Our structural analysis revealed distinctive features of GluN1a-3A NMDARs, prompting us to conduct an in-depth structural comparison with the more conventional NMDAR, GluN1-2B NMDAR, in both antagonist-bound inhibited and agonist-bound preactive states (*5*, *10*). Comparison of the CNQX-bound GluN1a-3A NMDAR with the antagonist-bound GluN1b-2B NMDAR (L689,560/SDZ220-040) indicated that the overall structures are similar, including domain swapping between the ATD and LBD layers (Fig. 4). To compare patterns of subunit arrangement, we calculated the center of mass (COM) of each ATD and LBD and measured the dimensions of GluN1a-3A NMDARs and GluN1b-2B NMDARs in agonist- and antagonist-bound forms (Fig. 4A; PDB code 6WHU = antagonist-bound/6WI1 = agonist-bound preactive state). The GluN1a-3A ATD dimers are substantially more separated than the GluN1b-2B ATD dimers, stemming from the characteristic of the ATD being highly mobile in the GluN1a-3A NMDAR (Fig. 4B). The dimensions of the LBD layer are similar except for the glycine-bound GluN1a-3A NMDAR, where the GluN3A LBDs rotate by ~80° relative to the CNQX-bound form. Such agonist-induced change does not occur in the GluN1b-2B NMDAR, again indicating that the glycine-induced GluN3A LBD rotation is a unique feature among NMDARs (Fig. 4C). The defined LBD orientations control the tension of the linker between the pore-forming M3/M3′ helices and the LBD, which regulates channel gating and can be presented by distances between the two gating ring residues at the tip of the linker (Fig. 4, D and E; GluN3A Glu^776^ and GluN2B Gln^662^ in spheres). For example, the distance for the GluN1b-2B NMDAR representing the preactive state has a longer inter-Gln^662^ distance (61.0 Å) than the antagonist-bound form (45.3 Å), indicating higher LBD-M3′ loop tension. The equivalent inter-Glu^776^ distance in the CNQX-bound GluN1a-3A NMDAR (47.2 Å) is similar to the antagonist-bound GluN1b-2B NMDAR (45.3 Å) (Fig. 4, D and E). The inter-Glu^776^ distance in the glycine-bound GluN1a-3A NMDAR is substantially shorter (23.5 Å), and its orientation differs from others, indicating little or no tension in the LBD-M3′ linker. Consequently, the channel gates are closed in both glycine-bound and CNQX-bound GluN1a-3A NMDAR structures.

### Disrupting the unique GluN1a-3A interface in the glycine-bound structure

**Fig. 5.**
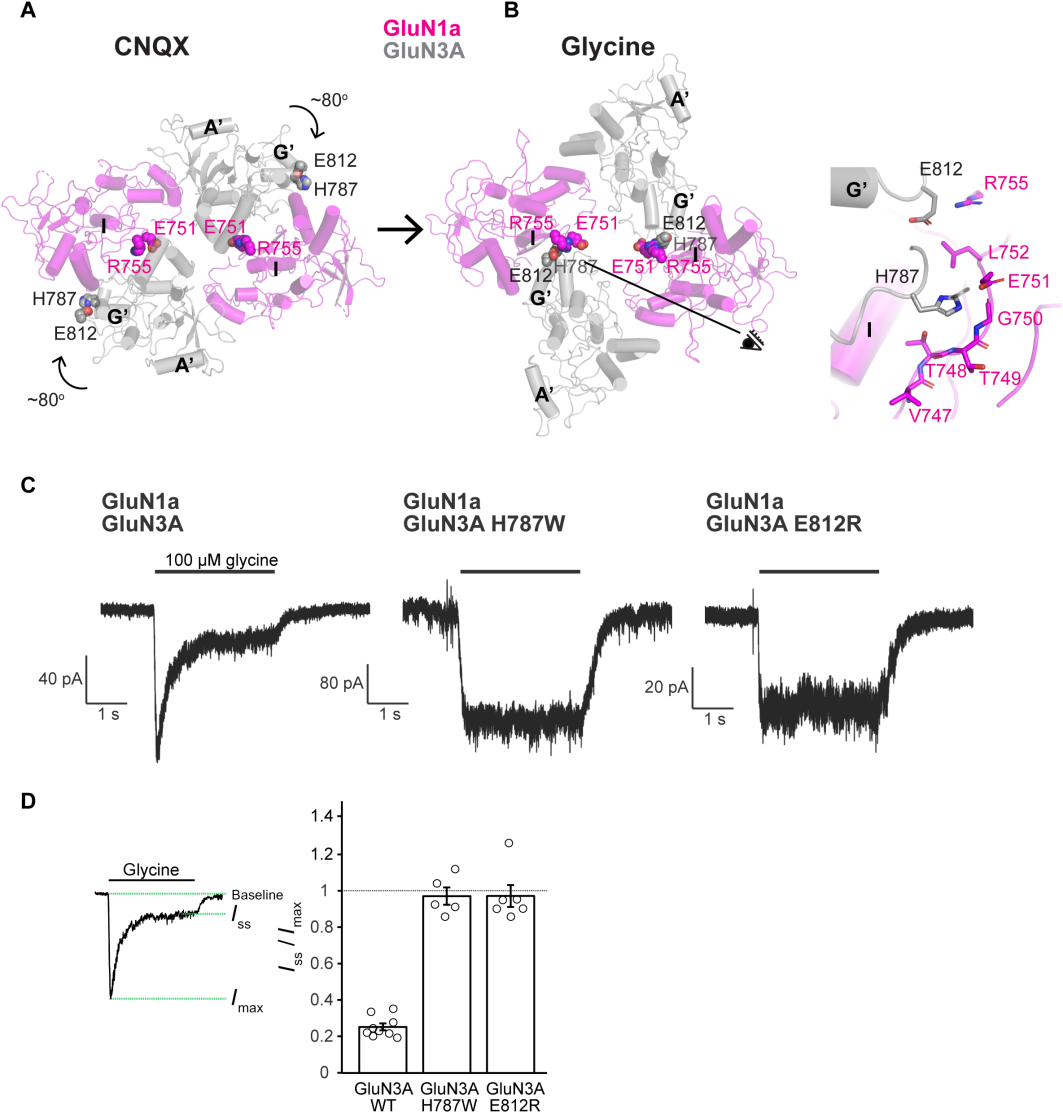
Site-directed mutations at the LBD layer disrupts desensitization. (**A** and **B**) CNQX-bound (A) and glycine-bound (B) GluN1a-3A NMDAR structures viewed from the top of the LBD layer. The rotation transitioning from the CNQX-bound conformation to the glycine-bound conformations results in formation of the new subunit interface around GluN3A His^787^ and Glu^812^ with GluN1a Glu^751^ and Arg^755^. Specific interactions are shown in the zoom-in panel (right) viewed from the “eye” symbol. (**C**) Whole-cell patch-clamp recording of WT, GluN3A His^787^Trp, and GluN3A Glu^812^Arg mutants, showing abolishment of desensitization in the mutant channels. (**D**) Extent of desensitization measured by *I*_ss_/*I*_max_. Error bars represent mean ± SE. Each point represents a single patch.

We hypothesized that the unique subunit arrangement of the GluN1a-3A NMDAR bound to glycine could reflect a desensitized state. This inference arises from the observation that the LBD-M3′ loop tension is minimal, and the channel pore remains closed, despite the LBDs adopting the close-cleft conformation induced by glycine binding (Fig. 4). To investigate this hypothesis, we directed our attention toward the inter-subunit interactions uniquely observed within the glycine-bound structure, in contrast to their absence in the CNQX-bound structure (Fig. 5). Specifically, two residues, GluN3A His^787^ and Glu^812^ around helix G′, are solvent-exposed in the CNQX-bound conformation (Fig. 5A), whereas they are proximal to the GluN1a residues around helix I, Glu^751^ and Arg^755^, to form the inter-subunit interface (Fig. 5B). For example, GluN3A His^787^ forms a polar interaction with GluN1a Gly^750^, and GluN3A Glu^812^ forms an electrostatic interaction with GluN1a Arg^755^ (Fig. 5B, right). We predicted that mutations at these two positions would destabilize this specific interface and alleviate desensitization of the GluN1a-3A NMDAR. Specifically, we introduced the mutations, His^787^Trp and Glu^812^Arg, into the full-length GluN3A and performed patch-clamp electrophysiology using HEK293 cells. We posited that introducing tryptophan at position 787 in GluN3A would create steric hindrance, preventing it from effectively interacting with GluN1a Gly^750^. Furthermore, we expected that introducing an arginine at position 812 in GluN3A would disrupt interactions with GluN1a Arg^755^, owing to charge repulsion.

In the WT GluN1a-3A NMDAR, glycine binding initiates channel activation, followed by robust desensitization, resulting in a steady-state current at approximately 20% of the peak current (Fig. 5, C and D). However, when glycine is applied to GluN1a-3A NMDAR harboring either the GluN3A His^787^Trp or Glu^812^Arg mutation, we observe no evidence of channel desensitization (Fig. 5, C and D). This result implies that destabilization of the unique GluN1a-3A subunit interface in the glycine-bound condition disfavors the process of desensitization and that the unique subunit arrangement of the glycine-bound GluN1a-3A NMDAR likely represents desensitization.

## DISCUSSION

**Fig. 6.**
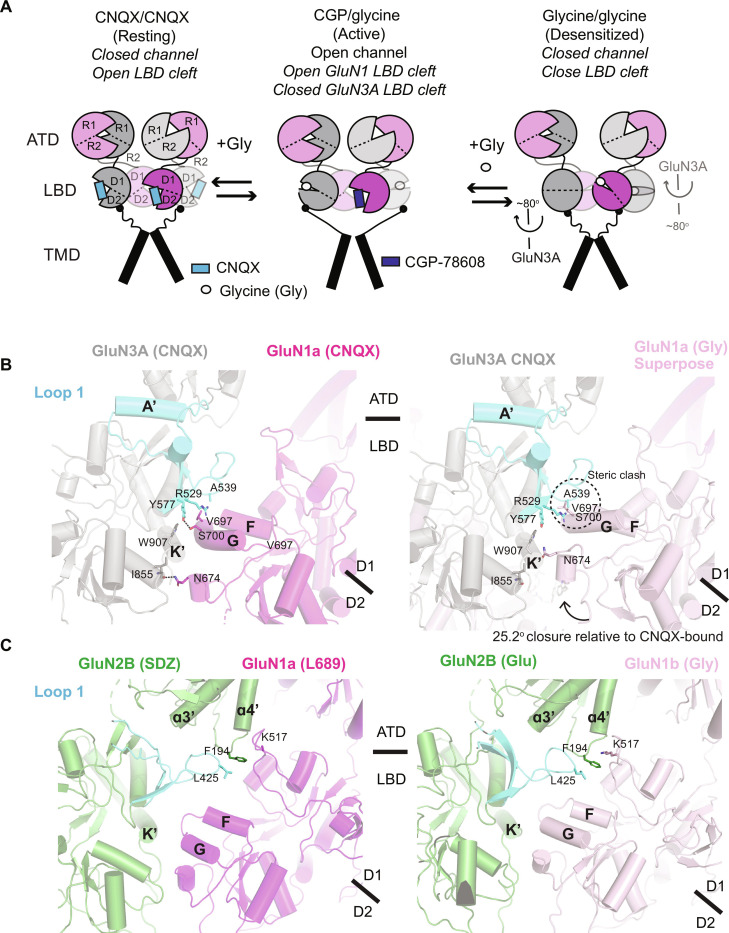
Mechanistic insights into unique features of GluN1-3A NMDAR. (**A**) Possible scheme of structural transition from resting (CNQX/CNQX-bound), active (CGP-70608/glycine-bound), and desensitized states (glycine/glycine-bound). While the active-state structure is not available, the open-cleft GluN1 LBD likely maintains the resting-state–like subunit arrangement. Occupation of GluN1 and GluN3A LBDs with glycine results in the ~80° rotation, resulting in desensitization. (**B**) Dimer of heterodimer interface at the LBD in the CNQX-bound structure (left). The closed-cleft GluN1a LBD was superposed; steric clash occurs between GluN3A loop 1 (cyan) and GluN1a helices F and G (right). There is no interaction between the LBDs and the GluN3A ATD at this site. (**C**) Equivalent dimer of dimer interface at the LBD of antagonist-bound (L689,560/SDZ220-040, PDB code: 6WHU, left panel) and agonist-bound (glycine/glutamate, PDB code: 6WI1) GluN1-2B NMDARs. The agonist binding does not result in steric clash with GluN2B loop 1 (cyan). Instead, GluN2B loop 1, ATD, and GluN1 LBD interact with each other to form a hub for allosteric coupling via residues such as GluN1a Lys^517^ and GluN3A Phe^194^ and Leu^425^.

Here, we unveiled the structures of the excitatory glycine receptor, GluN1a-3A NMDAR, revealing the unique pattern of subunit arrangement coupled with desensitization. We observed that glycine binding induces not only closure of the GluN1a and GluN3A LBD bi-lobes but also an additional ~80° rigid-body rotation of GluN3A LBDs, resulting in a previously unobserved receptor conformation (Fig. 6A and movie S1). Notably, desensitization was abolished when we introduced mutations to disrupt the subunit interface unique to the glycine-bound form, suggesting that this particular conformation represents the desensitized state.

This robust rotational movement of the LBDs has not been documented among the NMDAR family members. A somewhat analogous LBD rotational pattern has previously been observed upon glutamate binding to the homotetrameric kainate receptor, GluK2, where LBD rotation has been shown to contribute to receptor desensitization (*47*). Nonetheless, due to the relatively modest sequence similarities between GluK2 and GluN3A, standing at only 19.7% and 30% within the ATD and LBD regions, respectively, it is reasonable to assume that any common mechanistic similarities between the two are likely to be scarce.

An unusual feature of GluN1a-3A NMDAR is that GluN1-specific competitive antagonists act as potentiators instead of channel inhibitors (*19*, *20*, *29*). For example, CGP-78608 has high affinity toward GluN1 and normally inhibits GluN1-2 NMDARs, while it potentiates the GluN1-3A NMDAR current by 50- to 100-fold (*19*, *20*, *29*). A previous study showed that CGP-78608 traps the GluN1 LBD bi-lobe in an open cleft conformation (*9*). Our structures here allow us to infer that an open-cleft GluN1a LBD is a necessary prerequisite for GluN1a-3A NMDAR channel potentiation by GluN1-specific competitive antagonist. In the CNQX-bound form, interactions between the GluN3A and GluN1a LBDs at the dimer of heterodimer interface involve the F and G helices from GluN1a and the GluN3A loop 1 motif (Fig. 6B, left). However, superimposition of a glycine-bound GluN1a LBD reveals substantial steric clashes with the GluN3A loop 1, indicating that the closure of the GluN1a LBD is incompatible with the GluN3A LBD subunit orientation in the CNQX-bound form (Fig. 6B, right). Therefore, we speculate that glycine binding to GluN1a destabilizes the dimer of heterodimer interface, thus facilitating the rotation of the GluN3A LBD to avoid steric hindrance. In contrast, the open-cleft configuration of the GluN1 LBD prevents rotation of the GluN3A LBD due to specific inter-subunit interactions (Fig. 6B, left).

The interface equivalent to that found in GluN1-2B NMDAR differs from that in GluN1a-3A NMDAR. One apparent dissimilarity lies in the local architecture of GluN2B loop 1, which bears no sequence or structural similarity to GluN3A loop 1. The architecture of GluN2B loop 1 is such that it effectively avoids any steric clash when the GluN1a LBD cleft undergoes closure (Fig. 6C). Furthermore, the arrangement of GluN1 LBDs exhibits slight variations, notably because in GluN1-2B NMDAR and all other GluN1-2 NMDARs, GluN2 loop 1, GluN2 ATD, and GluN1 LBD interact cooperatively to govern inter-subunit and inter-domain interactions (Fig. 6C). This ATD-mediated interaction is absent in the GluN1a-3A NMDAR, potentially allowing greater flexibility in the movements of its subunits relative to one another. Moreover, considering the crucial role of ATD-LBD interactions in ATD-mediated allosteric modulation within GluN1-2 NMDARs, the absence of such interactions aligns with reports indicating that ATD plays a minimal role in functional regulation of the ion channel, as demonstrated through assays involving a GluN3A ATD-truncated construct (*18*). In summary, our study has provided insights into the structural and functional aspects of the GluN1a-3A NMDAR, shedding light on the fundamental reasons behind its unique functional characteristics compared to more typical GluN1-2 NMDAR family members.

## MATERIALS AND METHODS

### Expression and purification

Genes encoding human GluN1a (isotype without exon 5) and human GluN3A were cloned into a bicistronic expression vector under control of *Drosophila* Hsp70 promotors, as described previously (*30*, *31*). The GluN3A signal peptide was replaced with the signal peptide of the rat GluN2B subunit to further increase expression. For purification purposes, a Twin-Strep II tag was inserted immediately after the GluN3A signal peptide and a 1D4 epitope was added to the GluN3A C terminus. The GluN3A construct used for the cryo-EM study harbors residues from Gly^27^ to Ser^967^ with the Thr^675^Cys mutation. The GluN1a construct used for EM harbors residues Met^1^ to Gln^847^ with the Phe^810^Cys mutation. Baculovirus was generated following transfection of bacmid DNA into Sf9 cells, and 30 ml of amplified virus was used to infect 1-liter cultures of Sf9 cells grown to a density of ~500 × 10^4^ cells/ml in CCM3 medium (Cytiva). Following 48 hours of incubation at 27°C with shaking, cells were harvested by centrifugation at 4000 rpm for 10 min at 4°C (JA 4.2 rotor), suspended in cold tris-buffered saline (TBS) (20 mM Hepes, pH 7.5, 200 mM NaCl), pelleted in 50-ml conical tubes, and flash-frozen in liquid nitrogen.

Cells were suspended in HBS (200 mM NaCl, 20 mM Hepes pH 7.5) + 1 mM phenylmethylsulfonyl fluoride (PMSF) and lysed by two passes through an Avestin cell disruptor (~10,000 psi), and membrane material was collected by centrifugation at 40,000 rpm for 45 min at 4°C (Ti45 rotor). Membranes were solubilized in HBS + 0.5% LMNG (~10 ml per 1 g of membrane) for 2 hours at 4°C with stirring. Insoluble material was removed by centrifugation at 40,000 rpm for 45 min at 4°C, and the soluble fraction was loaded onto a StrepTactin Sepharose column (IBA Lifesciences). The column was washed with 10 column volume (CV) wash #1 [20 mM Hepes, pH 7.5, 200 mM NaCl, 0.05% digitonin (Millipore)], 20 CV wash #2 [wash #1 with 3 mM Mg-ATP (adenosine triphosphate) (Affymetrix)], and 10 CV wash #1 and eluted with 5 CV elution buffer (wash #1 supplemented with 3 mM desthiobiotin).

Protein was concentrated to ~1 ml in a 100-kDa Centricon (Millipore), and PMAL-C8 (Anatrace) was added at a ratio of 1 mg of protein to 5 mg of PMAL-8 and dissolved for 2 hours at 4°C by rotating. Bio-beads SM2 (Bio-Rad) were activated with methanol and washed extensively with water, and 75 mg was added to the protein mixture and mixed for 1 hour. A second batch of 75 mg of bio-beads was added, and the slurry was mixed overnight. Bio-beads were removed from the sample by passing the slurry over a Bio-Rad econo pack column. Protein was concentrated to 500 μl and separated on a Superose 6 10/300 column (Cytiva) in running buffer consisting of 20 mM Hepes (pH 7.5) and 200 mM NaCl. For CNQX-bound receptor, 30 μM CNQX (Tocris) was included in all purification steps starting from cell lysis. For glycine-bound receptor, 1 mM glycine was included in all purification steps. Unless otherwise stated, all chemicals were obtained from Sigma-Aldrich.

### Grid preparation and cryo-EM

Peak protein fractions were concentrated to ~7 mg/ml and then mixed with BS3 (Thermo Fisher Scientific) and GDN (Anatrace) to 1 mM and 0.1% final concentrations, respectively. The reaction was incubated on ice for 2 hours before 1 μl of tris (pH 8.0) was added to 10 mM final concentration to quench residual BS3. CF1.2/1.3 (Protochips), Quantifoil R 2/1 300 mesh [Electron Microscopy Sciences (EMS)], or UltrAUfoil R 1.2/1.3 Au 300 (EMS) grids were glow-discharged in a PELCO easiGlow instrument for 25 s under 15 mA. Grids were mounted in Vitrobot Mark IV (Thermo Fisher Scientific) set to 20°C, 80% humidity, blot force 7, and blot time 4.5 and blotted with 3.5 μl of protein before plunging into liquid ethane.

Data were collected on the Titan Krios (Thermo Fisher Scientific) at Cold Spring Harbor Laboratory operated at 300 keV using a Gatan K3 Summit direct electron detector coupled with a GIF quantum energy filter (Gatan) using a magnification of ×105,000 (0.856 Å/pixel). EPU software (Thermo Fisher Scientific) was used for image acquisition using a defocus range of −2.2 to −0.8 μm and a total dose ranging from 55 to 76.5 e^−^/Å over 30 frames. A total of five grids were used for reconstruction of the CNQX-bound receptor, and three grids were used for reconstruction of the glycine-bound conformation. Micrographs were processed in cryoSPARC (Structura Biotechnology) and processed using patch motion correction and contrast transfer function was estimated with patch CTF. Blob picker was used to generate an initial reconstruction, from which 2D averages were generated and used for template picking. Extracted particles were subjected to iterative rounds of multibody heterogeneous refinement to remove bad particle picks. Nonuniform refinement was used to generate high-resolution volumes. Models for GluN1a-3A were built based off previous cryo-EM or x-ray structures of GluN1 or GluN3A and fit into the cryo-EM density in UCSF Chimera. COOT was used to make manual adjustments before the model was refined against cryo-EM maps using Phenix real-space refinement.

### Electrophysiology

HEK293T cells were maintained in Dulbecco’s modified Eagle’s medium (DMEM) (Corning) + 10% fetal bovine serum (FBS) (Sigma-Aldrich) in an incubator set to 5% CO_2_ and 37°C. Cells were plated into wells of a six-well plate (Corning) and transfected at ~70% confluency with plasmids containing full-length human GluN1a or full-length human GluN3A at a ratio of 500 ng + 500 ng each, using Transit 2020 (Mirus Bio) according to the manufacturer’s instructions. After 24 hours of transfection, cells were gently resuspended and plated onto glass coverslips (VWR) and allowed to adhere for several minutes. Borosilicate glass micropipettes (Sutter Instruments) were pulled and polished to a final resistance of 2 to 5 megohms, backfilled with 110 mM d-gluconic acid, 110 mM CsOH, 30 mM CsCl, 5 mM Hepes, 4 mM NaCl, 0.5 mM CaCl_2_, 2 mM MgCl_2_, 5 mM BAPTA, 2 mM Na-ATP, and 0.3 mM Na-GTP (guanosine triphosphate), pH 7.35 with CsOH, and used to obtain patches in an external buffer composed of 150 mM NaCl, 4 mM KCl, 10 mM Hepes, 0.01 mM EDTA, 0.5 mM CaCl_2_, and 11 mM d-mannitol, pH 7.4 with NaOH. Whole-cell patches were lifted in front of a rapid solution exchanger (RSC-200, BioLogic) to perfuse cells with ligands and compounds. Data were collected on an Axopatch 200B amplifier (Axon Instruments), filtered at 2 kHz (Frequency Devices), and digitized with Digidata 1550B (Axon Instruments) using a sampling frequency of 10 kHz. Data were analyzed in ClampFit 11.0 software (Axon Instruments). Tau values were calculated by fitting the glycine response to a single-term exponential equation.
