# Supplementary material for: Structure and function of GluN1-3A NMDA receptor excitatory glycine receptor channel
Source: Sci Adv. 2024 Apr 10;10(15):eadl5952. doi: 10.1126/sciadv.adl5952 (PMC11006217; doi:10.1126/sciadv.adl5952)
Supplement: Supplementary file 1 — Figs. S1 to S6 Table S1 Legend for movie S1 [file sciadv.adl5952_sm.pdf]

Science Advances
AAAS

Supplementary Materials for

Structure and function of GluN1-3A NMDA receptor excitatory glycine receptor channel

Kevin Michalski and Hiro Furukawa

Corresponding author: Hiro Furukawa, furukawa@cshl.edu

Sci. Adv. **10**, eadl5952 (2024)
DOI: 10.1126/sciadv.adl5952

The PDF file includes:

Figs. S1 to S6
Table S1
Legend for movie S1

Other Supplementary Material for this manuscript includes the following:

Movie S1

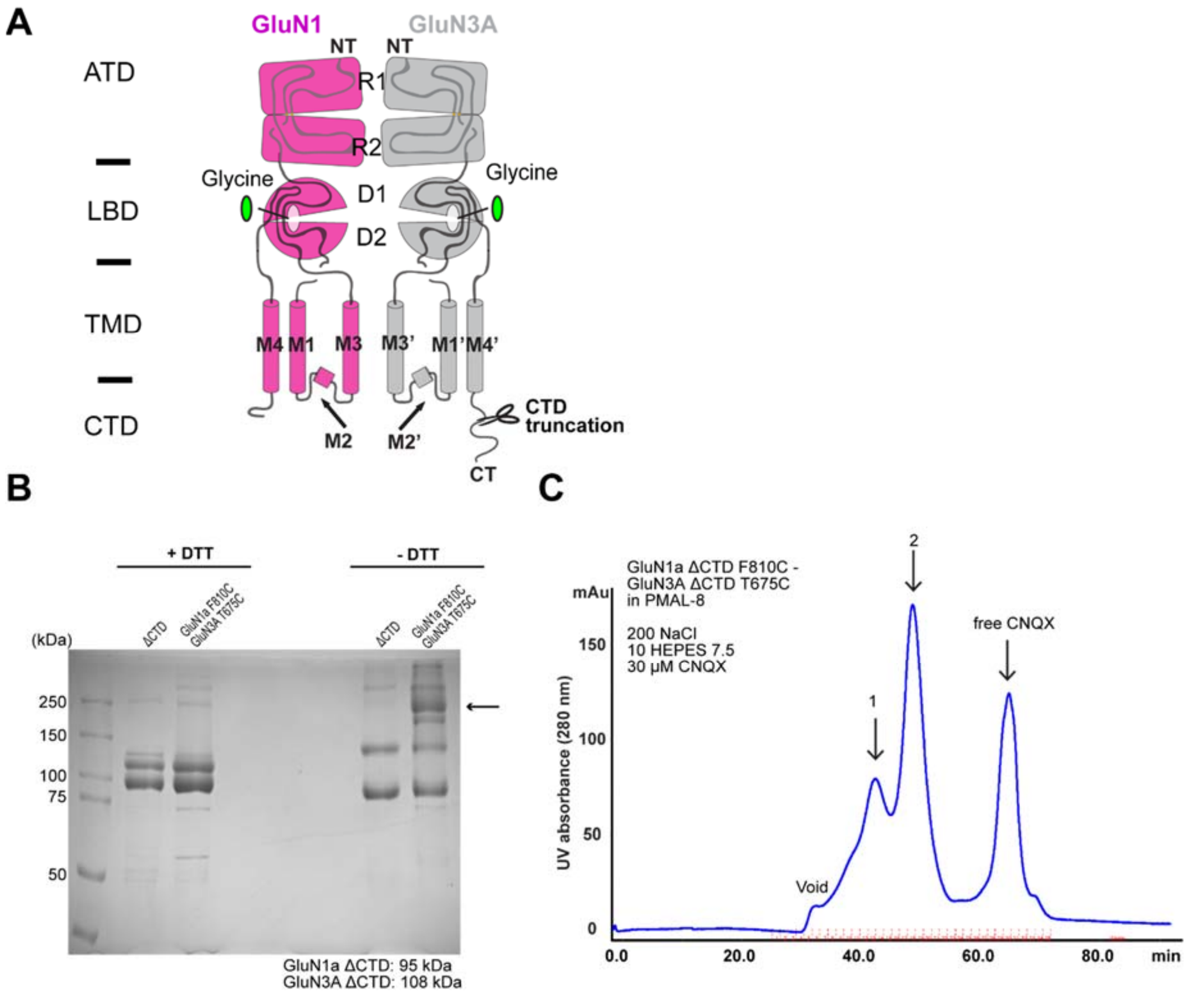

Fig. S1. Construct design and purification of GluN1a-3A receptors. (A) Schematic representation of GluN1a (magenta) and GluN3A (grey) depicting their modular structures, including the amino terminal domain (ATD, top), ligand binding domain (LBD, middle), and transmembrane domain (TMD, bottom). The C-terminal domain of GluN3A was truncated by 148 amino acids and terminated with Ser967, and the GluN1a subunit was truncated by 91 amino acids and terminated with Gln847. **(B)** SDS-PAGE gel of purified GluN1a-3A ΔCTD receptors with or without the stabilizing inter-subunit disulfide bridge formed by the GluN1a Phe810Cys and GluN3A Thr675Cys mutations. Proteins were mixed with sample buffer supplemented with or without 50 mM DTT, resolved by SDS-PAGE, and stained with Coomassie. Presence of a ~200 kDa band suggests formation of the inter-subunit crosslink. **(C)** Size exclusion chromatography of the GluN1a ΔCTD Phe810Cys - GluN3A ΔCTD Thr675Cys receptor. Proteins were purified over Streptactin Sepharose, exchanged into PMAL-8, and resolved on a Superose 6 10/300 column monitoring UV. Fractions from the main peak (arrow 2) representing monodisperse NMDARs were used for cryo-EM studies. Additional peaks indicated by arrows represent aggregated protein (arrow 1 and Void) and free unbound CNQX.

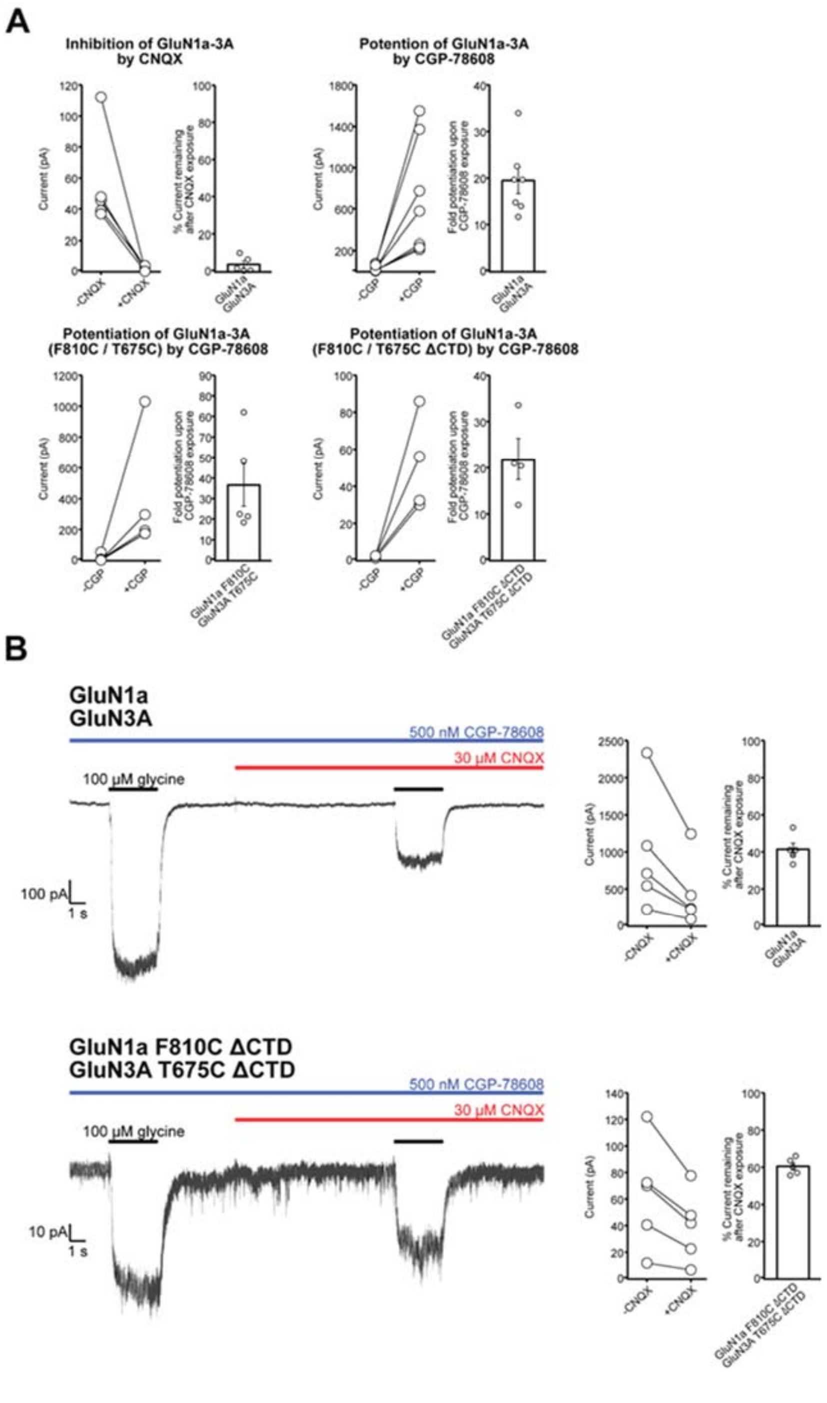

Fig. S2. Electrophysiological characterization of GluN1a-3A constructs. **(A)** Quantification of experiments performed in Figure 1. Peak currents before and after treatment with CNQX or CGP-78608 were plotted (left panels). Bar graphs measure the extent of inhibition by CNQX, or the fold potentiation upon CGP-78608 treatment. Bars represent the mean, each point represents a single patch, and error bars represent the SEM. **(B)** CNQX inhibition of WT GluN1a-3A and the cryo-EM construct under the tonic presence of CGP-78608. Patches were held in 500 nM CGP-78608 and glycine triggered currents were measured before and after a 10 s treatment with CNQX (left).

Peak currents were plotted before and after CNQX treatment, and bar graphs represent the extent of inhibition (right). Bars represents the mean, each point represents a single patch, and error bars represent the SEM.

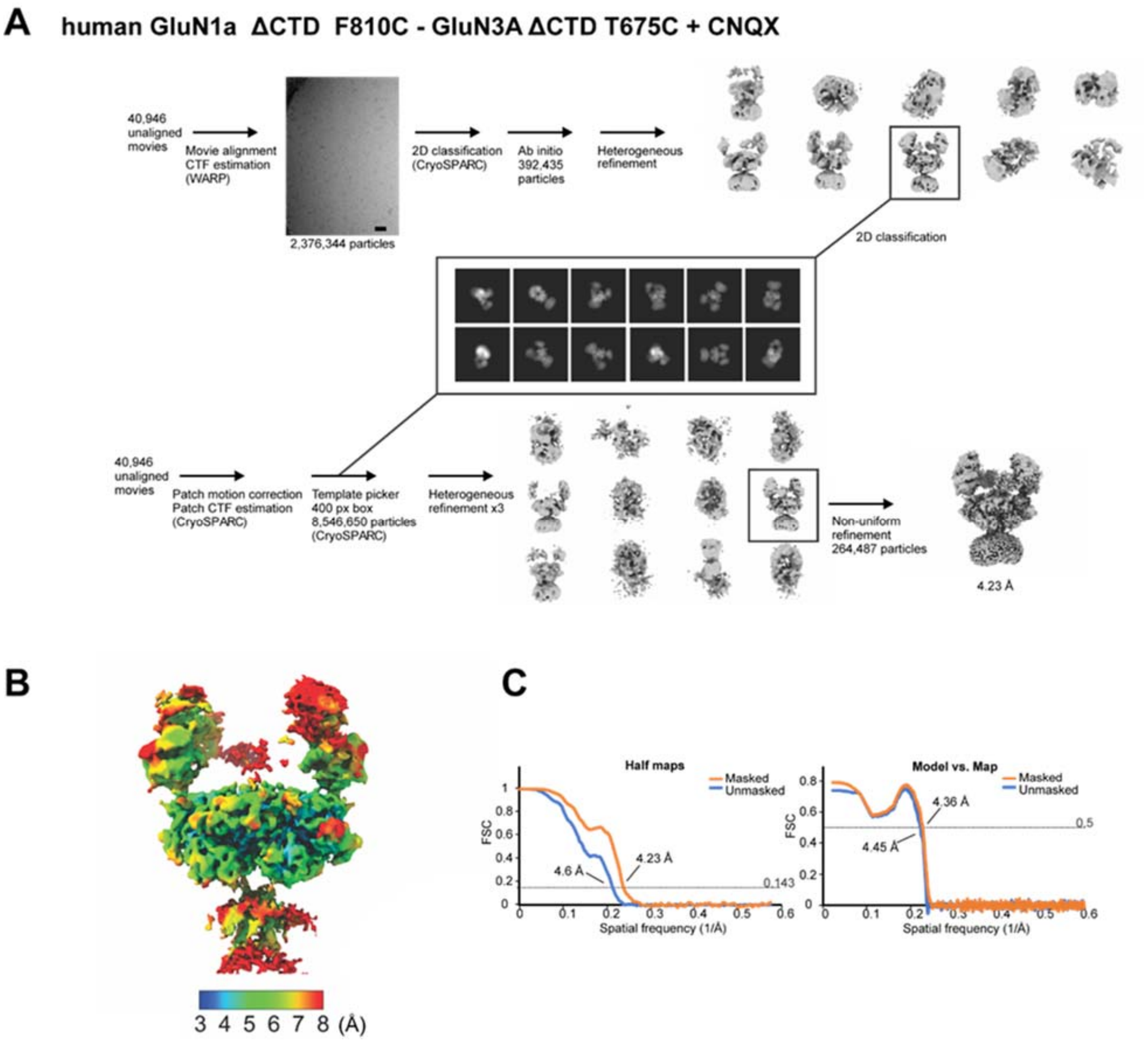

Fig. S3. Particle processing and cryo-EM data of GluN1a-3A in the presence of CNQX. (A) Micrographs and particles were initially processed on-the-fly to monitor ice quality and particle behavior. 2D averages were generated and used for template picking from micrographs processed separately in cryoSPARC. **(B)** Local resolution map of the final reconstruction. **(C)** Fourier shell correlations of the CNQX-bound receptor.

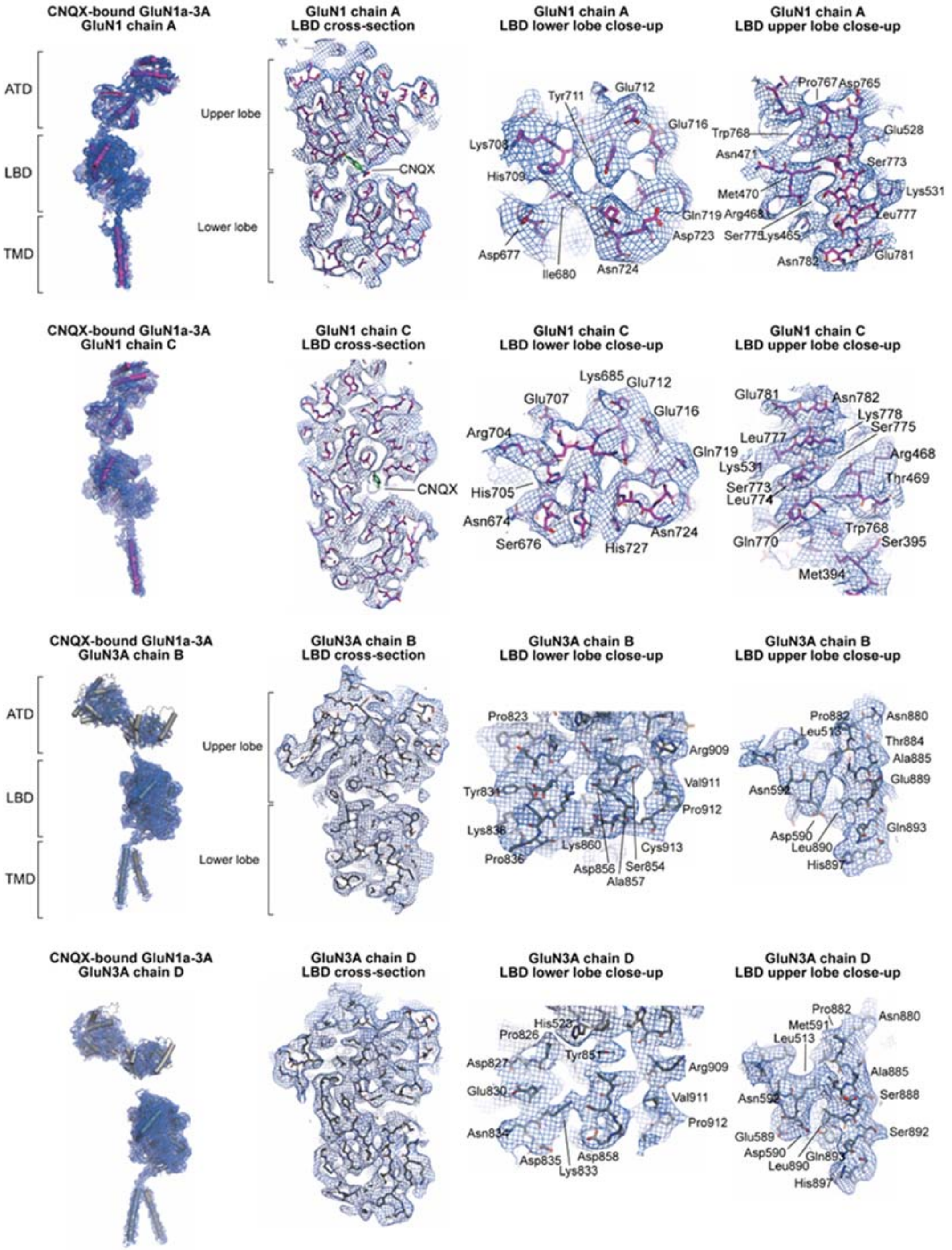

Fig. S4. Representative fit of CNQX-bound GluN1a-3A to the cryo-EM density. The cartoon model for GluN1a (chains A and C) are shown in magenta and cryo-EM density is shown as blue mesh. The cartoon model for GluN3A (chains B and D) are shown in grey. The overall fit of each respective chain is shown at different magnifications from the entire chain (left), a cross section of the LBDs (middle), and two examples of per-residue fit within the LBDs (right).

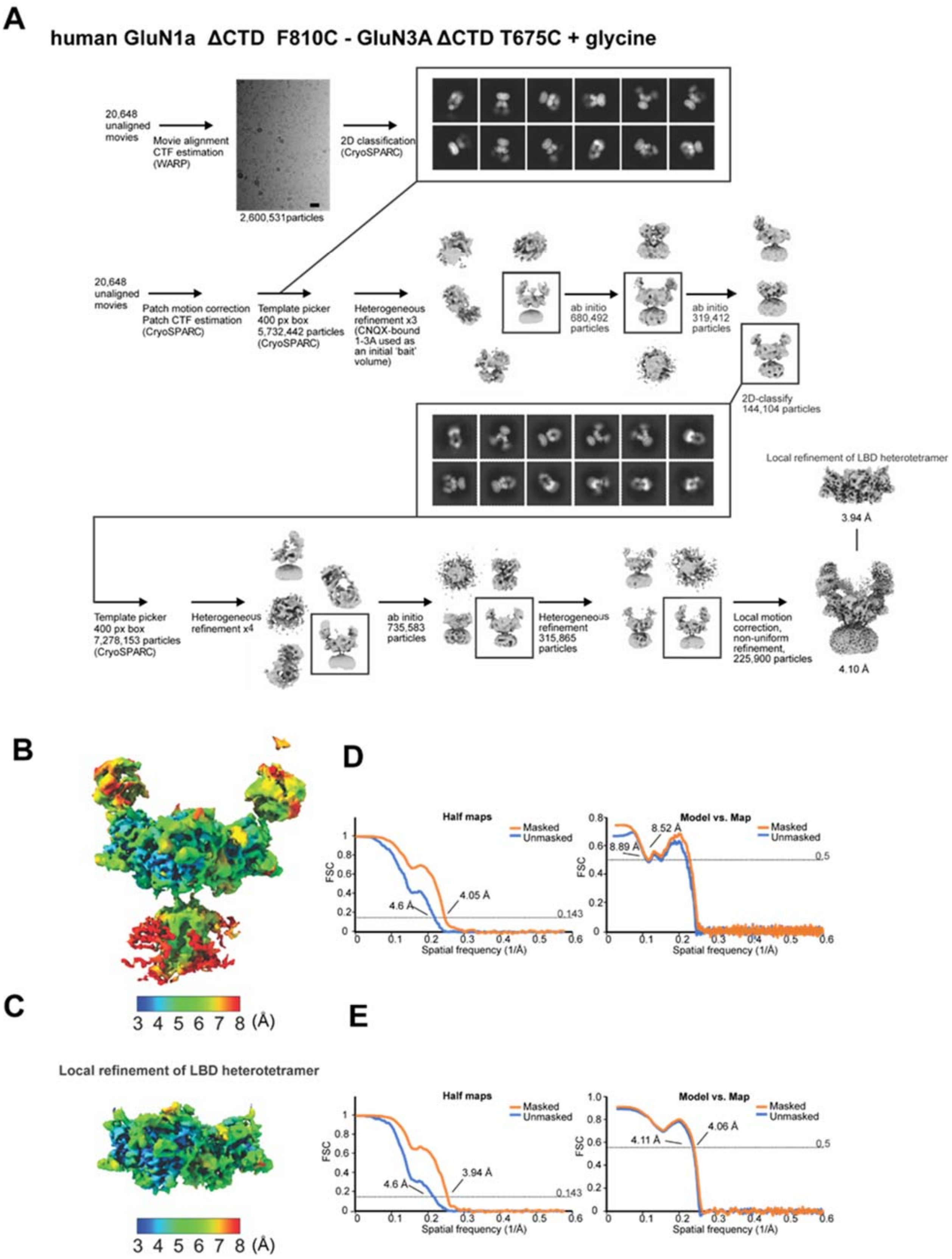
A
human GluN1a ΔCTD F810C - GluN3A ΔCTD T675C + glycine
20,648 unaligned movies
Movie alignment CTF estimation (WARP)
2,600,531particles
2D classification (CryoSPARC)
20,648 unaligned movies
Patch motion correction Patch CTF estimation (CryoSPARC)
Template picker 400 px box 5,732,442 particles (CryoSPARC)
Heterogeneous refinement x3 (CNQX-bound 1-3A used as an initial 'bait' volume)
ab initio 680,492 particles
ab initio 319,412 particles
2D-classify 144,104 particles
Local refinement of LBD heterotetramer
3.94 Å
Template picker 400 px box 7,278,153 particles (CryoSPARC)
Heterogeneous refinement x4
ab initio 735,583 particles
Heterogeneous refinement 315,865 particles
Local motion correction, non-uniform refinement, 225,900 particles
4.10 Å
B
C
3 4 5 6 7 8 (Å)
Local refinement of LBD heterotetramer
3 4 5 6 7 8 (Å)
D
Half maps
Masked
Unmasked
FSC
4.6 Å
4.05 Å
0.143
Spatial frequency (1/Å)
Model vs. Map
Masked
Unmasked
8.89 Å
8.52 Å
0.5
Spatial frequency (1/Å)
E
Half maps
Masked
Unmasked
FSC
4.6 Å
3.94 Å
0.143
Spatial frequency (1/Å)
Model vs. Map
Masked
Unmasked
4.11 Å
4.06 Å
0.5
Spatial frequency (1/Å)

Fig. S5. Particle processing and cryo-EM data of cryo-EM data of GluN1a-3A with glycine. **(A)** Micrographs and particle processing. Preliminary on-the-fly processing was used to generate 2D classes for template picking in cryoSPARC. An initial reconstruction from these picks was used to generate 2D classes for a second iteration of template picking. Multiple rounds of heterogeneous refinement and ab initio were used to clean the particles. **(B and C)** Local resolution of the overall glycine-bound GluN1-3A volume and the local refinement of the LBD heterotetramer. **(D and E)** Fourier shell correlations of the cryo-EM data and model validation.

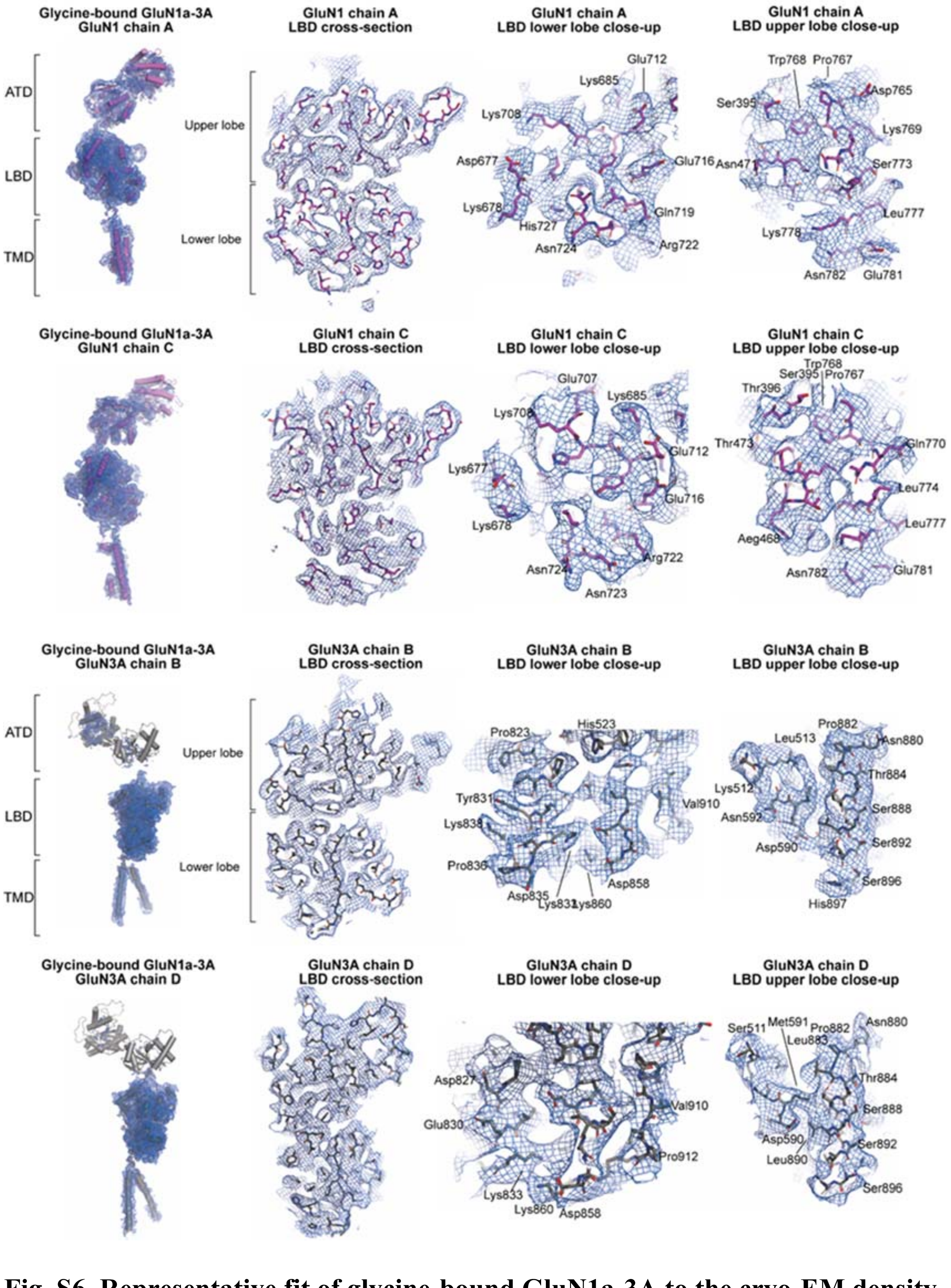

Fig. S6. Representative fit of glycine-bound GluN1a-3A to the cryo-EM density. The cartoon model for GluN1a (chains A and C) are shown in magenta and cryo-EM density is shown as blue mesh. The cartoon model for GluN3A (chains B and D) are shown in grey. The overall fit of each respective chain is shown at different magnifications from the entire chain (left), a cross section of the LBDs (middle), and two examples of per-residue fit within the LBDs (right).

Table S1. Cryo-EM data collection and refinement

	GluN1-3A +CNQX (EMDB-42520) (PDB 8USW)	GluN1-3A +glycine (EMDB-42522) (PDB 8USX)	GluN1-3A +glycine Local refinement (EMDB-42580) (PDB 8UUE)
**Data collection and processing**			
Microscope	Titan Krios	Titan Krios	Titan Krios
Camera	K3/counting	K3/counting	K3/counting
Magnification	105,00	105,00	105,00
Energy filter	Gatan	Gatan	Gatan
Energy filter slit width (eV)	20	10	20
Collection software	EPU	EPU	EPU
Voltage (kV)	300	300	300
Cumulative exposure (e–/Å^2)	55	76.5	76.5
Exposure rate (e–/Å^2/frame)	1.83	2.55	2.02
Defocus range (µm)	0.8-2.2	0.8-2.2	0.8-2.2
Pixel size (Å)	0.856	0.856	0.856
Symmetry imposed	C1	C1	C1
Number of micrographs	40,946	20,648	20,648
Initial particle images (no.)	8,546,650	7,268,153	7,268,153
Final particle images (no.)	264,487	225,900	225,900
0.143 FSC half map masked (Å)	4.23	4.05	3.94
0.143 FSC half map unmasked(Å)	4.6	4.6	4.6
**Refinement**			
Refinement package	Phenix	Phenix	Phenix
Initial model used (PDB code)	4kcd, 4kcc	2rc7, 7saa	2rc7, 7saa
0.5 FSC model resolution masked	4.36	8.52	4.06
(Å)	4.45	8.89	4.11
0.5 FSC model resolution unmasked			
(Å)			
Model resolution range (Å)	3.5-10	3.5-10	3-6
Map sharpening *B* factor (Å^2)	-173	-153.8	-137.8
Model composition			
Non-hydrogen atoms	17, 935	17,955	8,991
Protein residues	2,942	2,967	1,150
Ligands	2	0	0
CC map vs. model (%)	0.72	0.63	0.81
R.m.s. deviations			
Bond lengths (Å)	0.007	0.003	0.011
Bond angles (°)	0.741	0.604	1.308
Validation			
MolProbity score	2.14	1.95	2.3
Clashscore	12.16	9.11	15.99
Poor rotamers (%)	0.1	0.0	0.31
Ramachandran plot			
Favored (%)	90.22	92.64	88.45
Allowed (%)	9.44	6.85	10.67
Disallowed (%)	0.34	0.51	0.88
C-beta deviations	0.0	0.00	0.09
EMRinger Score	2.06	1.91	1.6
CaBLAM outliers (%)	4.78	4.72	6.89

Table S1. Cryo-EM data collection and model refinement statistics.

Movie S1. Conformation transition between CNQX-bound and glycine-bound GluN1a-3A NMDAR.
